# Enhancing Elderly Engagement: A Comprehensive Study on the Positive Impact of Robot‐Assisted Activities in Nursing Homes

**DOI:** 10.1002/alz70863_110548

**Published:** 2025-12-23

**Authors:** Arshia A Khan

**Affiliations:** ^1^ University of Minnesota Duluth, Duluth, MN USA

## Abstract

**Background:**

The integration of robots in nursing homes marks a transformative shift in elderly care, serving as both functional aids and sources of engagement and entertainment. Amid challenges tied to aging populations and limited resources, robots contribute to residents' well‐being by facilitating social interactions, providing cognitive stimulation, and offering recreational activities in nursing homes, representing a promising frontier in the evolution of care for aging populations.

**Method:**

In this study, a diverse group of residents aged 65 and older across multiple nursing homes engaged with a humanoid robot specially programmed for diverse activities, including joke‐telling, singing, dancing, playing games, and aiding with daily tasks. Utilizing a pre‐post design, baseline assessments were conducted before the robot's introduction, followed by regular post‐implementation evaluations using the Brief Introspection Mood Scale (BMIS), Montreal Cognitive Assessment (MOCA), and electrodermal activity (EDA) recorded through wearable sensors.

The study involved thorough training for nursing home staff on the robot's functionalities, and residents were gradually introduced to the robot through supervised interactive sessions, ensuring a smooth integration into the nursing home environment. Quantitative data from BMIS and MOCA underwent statistical analyses to discern patterns and changes over time, while EDA data were scrutinized for correlations with mood and cognitive assessments. Qualitative insights derived from resident and staff interviews, using thematic analysis, captured nuanced experiences.

**Results:**

The study confirmed the robot's effectiveness in engaging and entertaining residents, showcasing overwhelmingly positive outcomes. Residents consistently enjoyed enhanced mood and emotional well‐being, as indicated by substantial increases in positive affect according to BMIS scores. Quantitative analysis of MOCA scores revealed positive trends in cognitive functionality. Wearable sensors measuring EDA demonstrated heightened physiological arousal and positive emotional responses during residents' interactive sessions with the robot. Staff reported improved resident morale and observed the robot's effectiveness in creating a lively and interactive atmosphere within the nursing home.

**Conclusion:**

The results of this study affirm the multifunctional entertainment robot's positive impact on residents in nursing homes. From bolstering emotional well‐being to enhancing cognitive functions, the robot emerges as a promising tool for enriching the lives of elderly individuals in care settings.

